# Effect of a Community-Based Enhancement Program on Emergency Assistance and Marine Patient Transfer Coordination Competency Among Community Health Volunteers on the Remote Islands of Southern Thailand: Quasi-Experimental Study

**DOI:** 10.2196/80195

**Published:** 2026-03-31

**Authors:** Praditporn Pongtriang, Chulalak Kaewsuk, Pilaiporn Sukcharoen, Aranya Rakhab

**Affiliations:** 1 Department of Adult and Elderly Nursing, Faculty of Nursing, Suratthani Rajabhat University, Surat Thani, Thailand; 2 Department of Pediatric and Adolescent Nursing, Faculty of Nursing, Suratthani Rajabhat University, 272, Moo 9, Khunthalae, Muang, Surat Thani, 84100, Thailand, 66 77913375, 66 77913376

**Keywords:** knowledge, skill, training, technology, accident, health care, competency, marine transfer, remote Island, cardiopulmonary resuscitation, CPR

## Abstract

**Background:**

Currently, remote areas face problems accessing health services. Although emergency medical systems have a policy of pushing more rapid response units into these communities, they still have not covered many areas due to the distance and the lack of a specific system that fits the community context. The resulting delays to medical treatment after accidents and emergency illnesses in these areas thus increase the risk of severe symptoms, disability, and subsequent death.

**Objective:**

This study examines the effectiveness of a community-based enhancement program for emergency assistance and marine patient transfer coordination among community health volunteers (CHVs) on Phaluay Island, southern Thailand.

**Methods:**

This quasi-experimental study followed a 1-group pretest or posttest design. The research sample consisted of 30 CHVs selected through nonrandom purposive sampling. The research instruments were demographic questionnaires, knowledge and skill measures of emergency assistance, and a competency assessment of marine patient transfer coordination. The data analysis employed descriptive statistics, repeated measures analysis of variance, and the Bonferroni test, with statistical significance set at *P* less than .05.

**Results:**

The results revealed that the average scores on emergency assistance and marine patient transfer coordination knowledge were significantly higher (*P*<.001) after training compared to before, specifically at weeks 1 and 30. The average scores on emergency assistance and marine patient transfer coordination skills in weeks 1 and 30 in the CHVs were significantly higher (*P*<.001). The CHVs’ mean scores for emergency assistance and marine patient transfer coordination competency before (at week 1) and after training (at week 30) were significantly higher (*P*<.001).

**Conclusions:**

This research suggests that there is a need for policy advocacy by relevant agencies to further develop CHV competencies, which require continuous stimulation, monitoring, and reskilling to prepare these volunteers and other community members to respond effectively to emergencies in remote areas.

## Introduction

The issue of health care system access remains a significant concern in various parts of the world [1]. As a middle-income country, Thailand needs to develop its structural and foundational systems to support its growing population, just as much as its aging citizens [2]. However, Thailand faces obstacles to structural development, such as inadequate transportation [3]. This factor alone prevents various population groups from accessing medical care promptly, especially in remote and island areas [4]. Limited medical facilities that support potential treatment, as well as a shortage of health personnel who serve the people in these remote areas, pose further issues [5]. As in other Southeast Asian countries, there is a persistent shortage of medical personnel. Many countries in the region maintain a doctor-to-population ratio below the recommended standard of 10 per 10,000, especially in Cambodia, Indonesia, Laos, Myanmar, the Philippines, Thailand, Timor-Leste, and Vietnam. Cambodia reports the lowest ratio at 1.93 per 10,000, whereas Singapore achieves the highest ratio at 22.94 per 10,000 [6]. Although Thailand’s emergency medical system has a policy of pushing more rapid response units into the community, it may not cover all areas [7]. The resulting delays in timely medical treatment after accidents and emergency illnesses increase the risk of severe symptoms, disability, and subsequent death [8].

Phaluay Island, an island located in Thailand’s Surat Thani Province, has a population of approximately 500 people [9]. From the island, it takes more than 2 hours to reach the mainland by a small passenger boat. Emergencies such as motorcycle accidents and falls are common. As a result of its remoteness, Phaluay Island’s population, including tourists who visit the site, has been deeply affected by these and other forms of accidents and emergency illnesses, though there are difficulties in mitigating this issue. According to local health officials, the island has 1 subdistrict hospital and only 2 public health personnel for health support. This limitation can affect life and health conditions during an emergency. Moreover, no referral system exists to facilitate effective care and assistance for patients on Phaluay Island.

To receive health care, the village residents have had to use expensive private boats or their own small longtail boats, the latter of which may be at risk during emergency transport due to wind and waves. Depending on wave conditions, transport to sufficient treatment facilities can endure for more than 2 hours. This may result in patients receiving insufficient care before arrival. Based on the data on illnesses and patient referrals from 2020 to 2022, 25 emergency cases from Phaluay Island required referral to a mainland hospital; of those, only a small percentage utilized emergency medical services. Most patients were transported via a small boat. These limitations put patients at risk of not receiving care from staff during transport.

This makes it essential to develop knowledge [10] and various competencies related to initial care during health emergencies on Phaluay Island [11]. In such situations, community health volunteers (CHVs) play a crucial role in supporting primary health care through adequate emergency response [12,13], as learned through sufficient knowledge and skill training [14]. Recent research has revealed the positive impact of scenario training on rural CHVs in emergency response situations [15]. There is also a less common training program that integrates digital technology specifically into the island context. In addition, guidelines for assistance and emergency referrals for people on remote islands in Thailand are being made available to the public [16]. One such development enables volunteer leaders to provide care and assistance in emergencies by integrating communication technology, including ambulance operation centers (AOCs), into the marine patient referral system.

This research, therefore, focuses on integrating Phaluay Island’s AOC into its marine patient referral system to enhance patient care. This system has not yet been applied to small boat referrals, though utilizing technology can enhance this operational gap [17] and meet Thailand’s transformational needs [18]. This integration can serve as a guideline for strengthening and supporting individuals in remote areas to take care of themselves. It also involves coordinating with network partners to establish health care systems that enhance access to and promote equality in health care [19]. It may lead to the ultimate goal of people being safe, continuing to reduce the risk of disability and death rates.

Specifically, this study examines the effectiveness of a community-based enhancement program on emergency assistance and marine patient transfer coordination competency among CHVs on Phaluay Island, southern Thailand. We hypothesized that CHVs’ competency in providing emergency assistance and coordinating marine patient transfers after receiving this training will be statistically significantly higher than before the training (*P*<.05). This paper also highlights the process and impact of integrating the community context, technology-based approaches, and situated learning theory in the program’s development.

## Methods

This research followed a quasi-experimental, 1-group pre- or posttest design. It represents the third phase of the research and development project “Enhancement of Assistant and Referral Systems for Accidents and Emergency Illness on a Remote Island: Phaluay Island Safety Model.”

### Research Sample

#### Study Participants

To determine the effectiveness of the primary care knowledge and competency development program, people living on Phaluay Island were recruited as CHVs via nonrandom purposive sampling. A total of 30 people were recruited based on the following criteria: 18 years of age or older, no physical or visual limitations, no congenital diseases such as heart disease or asthma, willing to participate in the entire research process, and the ability to read and write in Thai. All participants were recruited and contacted by local health personnel to participate in this research.

#### Sample Size Calculations

This research compared the volunteers’ mean scores on knowledge and skills assessments. The researchers calculated the sample size based on a previous study that examined the effect of simulation training on enhancing nursing students’ perceptions to engage patients’ families in treatment plans [20]. With a power (*Z*β) of 80% (0.84), a significance level (*Z*α) of 0.05 (1.96), a mean difference of 1.1 (SD1 0.6 and SD2 0.3), k of 1 group, and statistical significance of 0.05, the sample size obtained for this study was 22 cases. To counter the chance of participants dropping out of the research, the researchers increased the sample size to 30 according to Cohen formula (n=22 cases) [21]:


$$Effect\;size=d_{\text{pools}}=\frac{m_{1}-m_{2}}{{SD}_{1}+{SD}_{2}}=f^{2}=\frac{d^{2}}{2k}=\delta^{2}=0.744$$

$$n=\frac{{\left(Z_{\alpha /2}+Z_{\beta }\right)}^{2}\times 2\sigma^{2}(1-\rho )}{\delta^{2}}$$

$$n=\frac{\left(1.96+0.84{)}^{2}\times 2(0.9{)}^{2}(1-0.05\right)}{(0.744{)}^{2}}$$

### Research Instruments

#### Training and Data Collection

The research instruments consisted of 2 parts—the community-based enhancement program and the knowledge and skill measure of emergency assistance and marine patient transfer coordination competency. First, the community-based enhancement program utilized situated learning theory [22] as a framework for developing training activities. This theory focuses on learning as participation through social interaction and a community of practice in which the individual shares and learns together. The program activities are based on the elements of activities, artifacts, identities, and relationships.

These elements were intended to develop the sample group’s knowledge and skills in providing basic health assistance to patients and referring marine patients. The details of the program are as follows: (1) organized activities, including lectures, demonstrations, reverse demonstrations, and practice simulations, developed CHVs’ diverse knowledge and skills; (2) artifacts involved the integration of AOC technology into potential development of the patient marine transfer support; (3) the program utilized 4 emergency simulation situations consistent with the Phaluay Island community’s identity and way of life to promote the application of available resources; and (4) emphasis on relationships promoted learning together in a group. The training program covered 5 operational stations: station 1, covering symptom assessment and first aid; station 2, on resuscitation and automated external defibrillator (AED) use; station 3, introducing maritime rescue and referral methods; station 4, concerning requests for assistance and communication; and station 5, for the implications of AOC system technology (Table 1). The development of these 5 station activities was underpinned by the elements of situated learning theory, which enhanced competency, knowledge, and skills in initial assistance for emergency patients, as well as the referral of marine patients from remote islands.

**Table 1.** The 5-station training program based on situated learning principles.

Station	Learning objectives	Duration (min)	Learning methods	Expected competencies
1. Symptom assessment and first aid	Conduct rapid assessments of emergency symptoms using a structured methodology. Identify clinical red flags and determine criteria for urgent referral. Provide immediate first-aid interventions appropriate for common emergencies encountered in the island context.	50	Instructional strategies included realistic scenario briefings, guided practice with authentic cases, peer coaching, facilitator feedback, and integration of local contextual cues.	Expected outcomes are accurate primary assessments, effective prioritization, safe first-aid interventions, and timely escalation decisions.
2. Resuscitation and AED^a^ use	Demonstrate high-quality cardiopulmonary resuscitation in accordance with American Heart Association guidelines. Operate an AED correctly, including accurate pad placement, completion of safety checks, and appropriate shock delivery. Coordinate team roles effectively during resuscitation, functioning as either a team member or leader.	60	Practice skills through simulation activities, team-based scenarios with defined roles, ongoing coaching, and structured debriefing sessions that focus on decision-making within time and distance limitations.	Competencies include proper execution of cardiopulmonary resuscitation steps, effective chest compressions, safe and accurate use of an AED, clear team communication, and rapid response.
3. Maritime rescue and referral procedures	Demonstrate safe maritime rescue principles and effective patient stabilization techniques for sea transfer. Prepare patients for referral, including appropriate positioning, monitoring, and securing. Utilize a standardized referral pathway for patient transfer from remote islands, specifying contact persons, timing, and required information.	60	Contextual demonstrations of boat and shore transfer workflows, task-based learning activities, and hands-on equipment handling.	Competencies include safe patient handling and movement, implementation of stabilization steps, risk mitigation for weather and sea conditions, accurate referral preparation, and completion of performance checklists with scenario-based decision points.
4. Requesting assistance and emergency communication	Demonstrate effective communication with dispatch and health services. Deliver complete and accurate information to support decision-making processes.	50	Role-play exercises involving caller, dispatcher, and team roles; both scripted and unscripted communication drills; structured feedback on clarity and completeness; guided reflection on authentic community communication barriers.	Demonstrate clarity, completeness, and timeliness in communication; route requests appropriately; confirm and close communication loops; and achieve proficiency as measured by a communication rubric and call simulation.
5. Implementation of technology and the AOC^b^ system for marine transfer	Demonstrate understanding of AOC functions, including coordination, dispatch, tracking, and documentation. Apply AOC-supported workflows, such as request initiation, status updates, and referral coordination.	50	System walk-through, hands-on practice with demonstration interfaces and workflows, case-based discussions on changes introduced by AOC, troubleshooting exercises, and reflective debrief sessions.	Competencies include accurate data entry and reporting, adherence to the correct workflow steps, timely updates and escalations, handling connectivity limitations, and successful completion of a task checklist.

a AED: automated external defibrillator.

b AOC: ambulance operation center.

**Figure 1.**
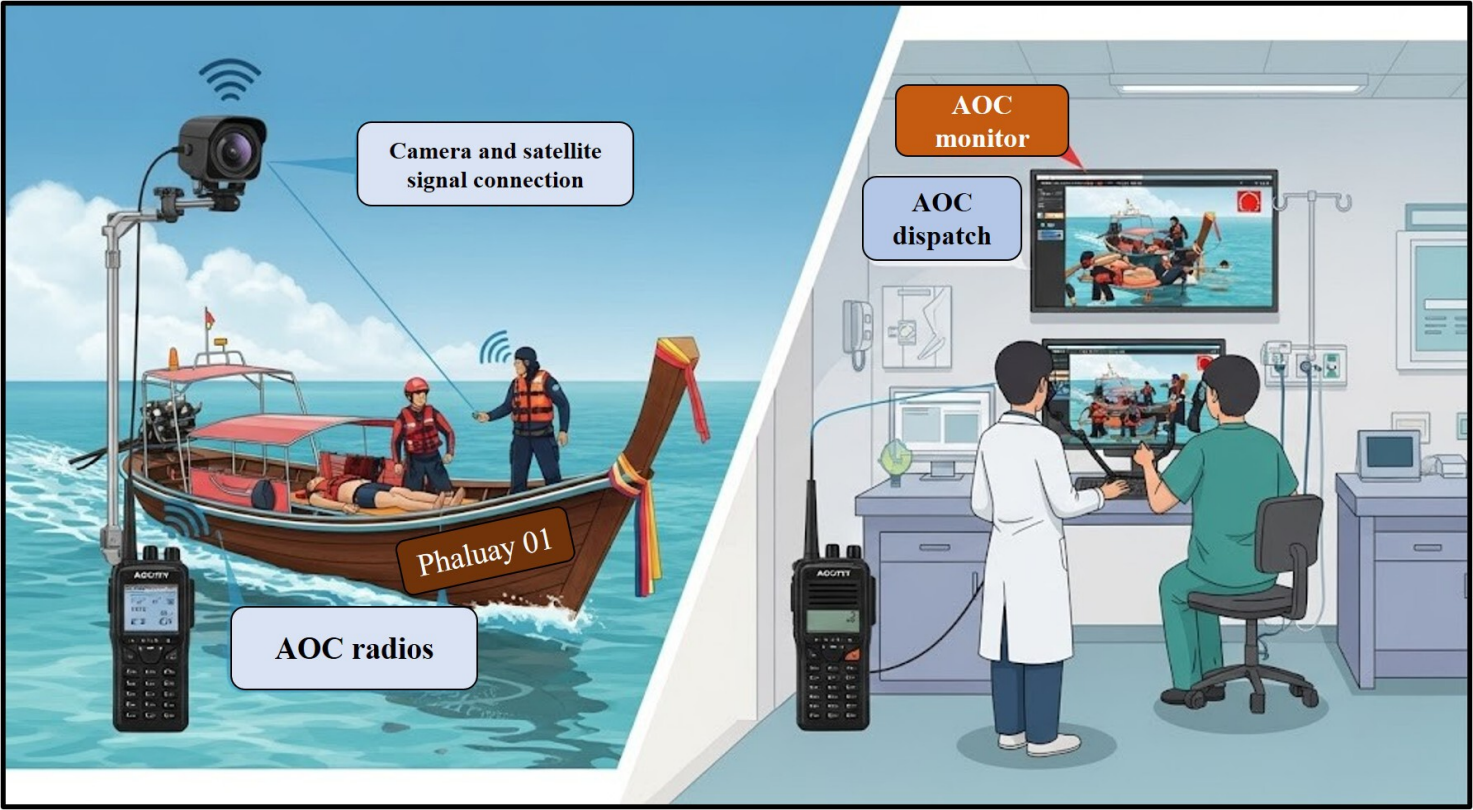
Ambulance operation center (AOC), generated using Google Gemini (artificial intelligence image generator), 2025 [24].

The program was conducted in 2 phases (30 weeks apart) for a total activity period of 12 hours. The training program included two components: (1) knowledge enhancement regarding first aid, basic life support (BLS), AED use, AOC, and communication in emergencies, and (2) skill development, consisting of demonstrating and then returning to demonstrate first aid, BLS, AED use [15,23], AOC implementation, communication activities, and maritime transfer. Activities of this phase fell into one of the following categories:

- Activities focusing on knowledge development over two and a half lecture hours to educate on fundamental assistance issues, such as symptom assessment, requesting help (emergency call number 1669), and communicating with the emergency medical system
- Workshop activities (1 h) on basic communication and assistance tools connecting to the AOC system
- Activities to develop competency in initial assistance and maritime referrals, such as condition assessment, movement, communication, and symptom monitoring, with the sample divided into groups in initial assistance exercises of 4 hours and 30 minutes each
- Activities to restore knowledge and skills in simulations (4 h each) in week 30
- An AOC equipment setup activity, as supported by TELY 360, demonstrated how to integrate the equipment into marine patient referral systems. This satellite signal connection device is used to track coordinates and locations during patient transfers, a common practice in secondary and tertiary health service facilities in Thailand. In the context of remote islands, the AOC was the first contact that the island had with the mainland. In addition, AOC devices, including radios to coordinate patient details with the operation center at Donsak Hospital and camera equipment to monitor patient status and referral teams (eg, CHVs and family caregivers), can be installed on the subject when a ready-to-use referral is available. This technological integration is a crucial component that enables the destination hospital to guide the referral team on board the ship during patient transport. The AOC device was placed in a portable box (as shown in Figure 1), and a designated, ready-to-use storage point was established for emergency patient transport in key areas, as agreed upon by the community.

The second part of the research instruments used for data collection were demographic characteristic questionnaires, the questionnaire of knowledge measurement, and the questionnaire of skill measurement, which are described as follows.

Demographic characteristic questionnaires gathered data on the volunteers’ age, occupation, and education level; number of experiences providing basic health care assistance to victims of accidents or emergency illnesses; experience in helping with marine patient transport; and BLS experience.

The researchers developed a multiple-choice knowledge measure of emergency assistance and marine patient transfer coordination competency among the CHVs based on a literature review of relevant research. Each question had 2 answer options (“yes” or “no”), with 1 point given for a correct answer and 0 points for a wrong answer. There were 15 questions, leading to possible total scores of 0 to 15 points. This questionnaire involved evaluating the situation, emergency notification, and first aid for patients who met with an accident in situations such as poisonous animal bites, hemostasis, basic immobilization, and BLS.

The researchers also derived a skill measure of emergency assistance and marine patient transfer coordination competency among CHVs based on a literature review. The measurement had a 3-level rating scale. The program instructors performed a skill assessment during practice activities by selecting 1 answer that matched each participant’s practice status: a complete practice yielded 2 points, an incomplete practice 1 point, and nonpractice 0 points. Participants could receive a total score between 0 and 36 on this 18-item questionnaire. This skill measurement focused on symptom assessment, communication, first aid, and BLS.

To evaluate assistance competency and marine patient transfer coordination, the researchers further assessed the volunteers’ total knowledge and practical skills scores, with scores ranging from 0 to 51 points.

#### Instrument Validity

Five qualified experts examined the research instruments to determine whether their content validity ratio values exceeded 0.99, except for the following exceptions: item 7 of the demographics instrument, item 2 of the knowledge measure, and items 4 and 8 of the skill measure. The experts provided improvements, and the researchers implemented them accordingly. Considering the questionnaires’ content validity index (CVI) results, the knowledge measure had a CVI of 0.98, and the skill measure had a CVI of 0.97. The researchers then administered each questionnaire to a pilot group of 30 people with characteristics similar to the sample. The reliability of the knowledge measure was also examined using the Kuder Richardson 20, which yielded a value of 0.737, while the skill measure yielded a Cronbach α coefficient of 0.964.

### Data Collection

**Figure 2.**
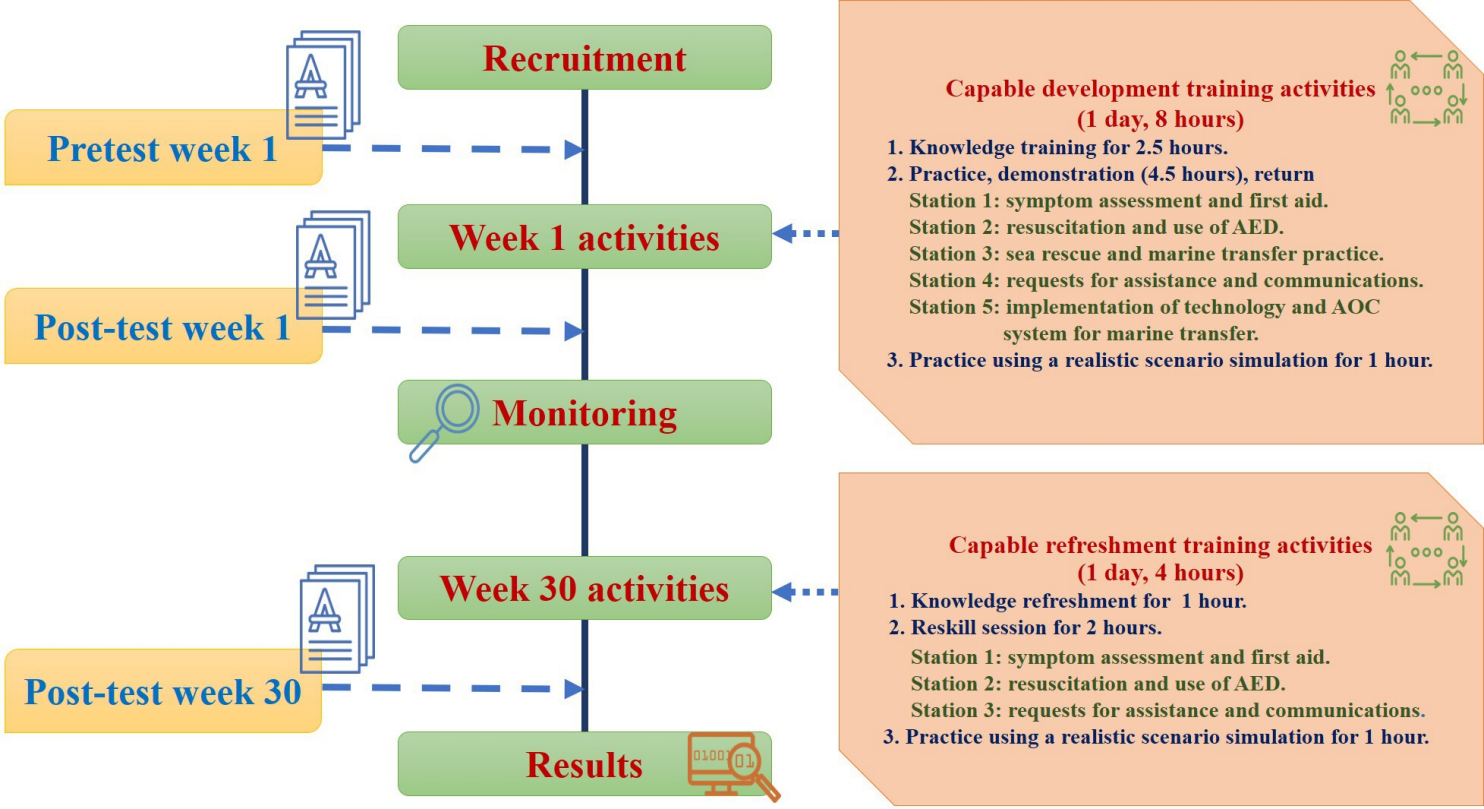
Data collection protocol. AED: automated external defibrillator; AOC: ambulance operation centers.

This research, in particular, was conducted between March and October 2024. After receiving ethics committee approval, the researcher proceeded to recruit the sample group to participate in the research process for a total of 30 weeks. The sequence of the quasi-experimental research process is outlined in Figure 2.

### Data Analysis

The demographic data were analyzed using descriptive statistics such as frequency, mean, percentage, and SD. The inferential statistics repeated measures ANOVA and the Bonferroni test were used to compare the mean differences in emergency assistance and marine patient transfer coordination knowledge, skills, and competencies among the volunteers before and after training at week 1 and week 30, respectively, with statistical significance set at *P*=.05.

### Ethical Considerations

This project was approved by the Human Research Ethics Committee of the Surat Thani Provincial Health Office (approval STPHO2023-318). The researchers followed the principles of the Belmont Report to ensure the protection of the volunteers regarding respect for persons, beneficence, and justice (National Commission for the Protection of Human Subjects of Biomedical and Behavioral Research, 1979) [25]. The volunteers received sufficient information before deciding to participate in the project and signing the consent form. The participants could withdraw from the project at any time without penalty or adverse effect. The participants’ information, including names and initials, was anonymized. All results will be presented and published for academic purposes and will be deidentified. The data and materials relevant to the participants will be stored in a secure room and destroyed 5 years after the first day of data collection. The participants in this study received a cash payment of 100 Thai baht per person for their time. They were also provided with one lunch (120 Thai baht; an exchange rate of US $1=32.6 Thai baht is applicable) and two snack breaks (30 Thai baht each). The total value provided was 280 Thai baht per participant.

## Results

From the preliminary analysis of the 30 CHVs, the sample group’s information was complete, and the data from 25 cases were analyzed because of 5 participants withdrawing from the project for personal reasons. The results showed that the sample had an average age of 48.20 (SD 13.43) years, and most individuals worked as freelancers. Forty percent (10/25) were involved in CHV work, while the majority had no experience in emergency aid (17/25, 68%), as shown in Table 2.

**Table 2.** Volunteer demographic data and emergency assistance experience (N=25).

Demographic data	Values
Career, n (%)	
Government officer	1 (4)
Farmer	3 (12)
Fisherman	1 (4)
Freelancer	10 (40)
State enterprises	2 (8)
Own business	8 (32)
Education level, n (%)	
Grade 6	15 (60)
Junior high school	4 (16)
Senior high school	3 (12)
Undergraduate	3 (12)
Role in the community, n (%)	
Community health volunteer	10 (40)
Community leader	2 (8)
Security officer	1 (4)
No specific role	12 (48)
Experience with emergency assistance, n (%)	
No	17 (68)
Yes	8 (32)
Age (y), range; mean (SD)	20-69; 48.20 (13.43)
Frequency of emergency assistance experience, range; mean (SD)	0-10; 1.52 (3.22)
Frequency of the marine patient transfer experience, range; mean (SD)	0-10; 1.53 (3.17)
Frequency of emergency calls experienced, range; mean (SD)	0-10; 0.64 (2.07)

A repeated measures ANOVA (n=25) revealed significant within-subject effects for all knowledge, skill, and competency. Knowledge scores increased significantly over time, *F*_(1, 24)_=41.379, *P*<.001, with a large effect size (*η*^2^*p*=.633; estimated mean, 95% CI 8.782‐10.312). Skill scores change significantly, *F*_(1, 24)_=58.532, *P*<.001, with a large effect size (*η*^2^*p*=.709; estimated mean 95% CI 23.815‐25.012). Competency scores also demonstrated significant improvement, *F*_(1, 24)_=37.755, *P*<.001, with a large effect (*η*^2^*p*=.611; estimated mean 95% CI 32.817‐35.103), as shown in Table 3.

**Table 3.** The variance of the variable within the training group for knowledge, skill, and competency (N=25).

Variable	SS^a^	MS^b^	*F* test (*df*)	*P* value	*η* ^2^ *p* ^c^	95% CI
Knowledge (error)	66.667 (38.667)	66.667 (1.611)	41.379 (1, 24)	<.001^d^	0.633	8.782-10.312
Skill (error)	499.280 (204.720)	499.280 (8.530)	58.532 (1, 24)	<.001^d^	0.709	23.815-25.012
Competency (error)	126.960 (257.880)	126.960 (10.745)	37.755 (1, 24)	<.001^d^	0.611	32.817-35.103

a SS: type III sum of squares.

b MS: mean square.

c 𝜂2𝑝: partial eta squared (effect size).

d Significant at *P*<.001.

The average scores on emergency assistance and marine patient transfer coordination knowledge before and after training, specifically at weeks 1 and 30, revealed that the CHVs’ level of knowledge was significantly higher (*P*<.001). When comparing pairs using the Bonferroni method, the average knowledge score before week 1 and before the formal start of the knowledge training likewise showed a statistically significant increase (*P*<.001). Between week 30 (after the program took place) and before the knowledge training began, there was also a statistically significant increase (*P*<.001). However, following the trials in week 1 and week 30, there were no statistically significant differences (*P*=.39), as shown in Table 4.

**Table 4.** Average scores on emergency assistance and marine patient transfer coordination knowledge before and after training (N=25).

Time	Score, mean (SD)	Before training	Week 1, MD^a^ (*P* value^b^)	Week 30, MD (*P* value^b^)	*P* value^c^
Before training	7.36 (2.039)	—^d^	3.52 (<.001)	3.04 (<.001)	<.001
Week 1	10.88 (2.369)	—	—	–0.48 (.39)	—
Week 30	10.40 (2.432)	—	—	—	—

a MD: mean differences (time 2 – time 1).

b Bonferroni test for pairwise comparisons.

c Repeated measures ANOVA for test of within-subject effects.

d Not applicable.

The average scores on emergency assistance and marine patient transfer coordination skills in weeks 1 and 30 in the CHVs were significantly higher (*P*<.001). When comparing pairs using the Bonferroni test in week 1 and before the training, the mean skill scores were statistically significantly different (*P*<.001). In week 30 and before the training, there was also a statistically significant difference in the skill level (*P*<.001). After the trials in weeks 1 and 30, the CHVs’ average skill levels were significantly different (*P*=.005), as shown in Table 5.

**Table 5.** Average scores on emergency assistance and marine patient transfer coordination skills before and after training (*N*=25).

Time	Score, mean (SD)	Before training	Week 1, MD^a^ (*P* value^b^)	Week 30, MD (*P* value^b^)	*P* value^c^
Before training	21.00 (1.871)	—^d^	3.92 (<.001)	6.32 (<.001)	<.001
Week 1	24.92 (1.631)	—	—	2.4 (.005)	—
Week 30	27.32 (3.579)	—	—	—	—

a MD: mean differences (time 2 – time 1).

b Bonferroni for pairwise comparisons.

c Repeated measures ANOVA for test of within-subjects effects.

d Not available.

The average scores on the CHVs’ overall emergency assistance and marine patient transfer coordination competencies before and after training, specifically in weeks 1 and 30, showed a significantly higher competency mean score among the CHVs (*P*<.001). Upon pair comparison using the Bonferroni test, the competency mean scores at week 1 and before training showed a statistically significant difference (*P*<.001). After the experiment at week 30 and before training, the competency levels were statistically significantly different (*P*<.001). Weeks 1 and 30 were also statistically significantly different (*P*=.006), as shown in Table 6.

**Table 6.** Average scores on overall emergency assistance and marine patient transfer coordination competencies before and after training (N=25).

Time	Score, mean (SD)	Before training	Week 1, MD^a^ (*P* value^b^)	Week 30, MD (*P* value^b^)	*P* value^c^
Before training	28.36 (0.568)	—^d^	7.44 (<.001)	9.36 (<.001)	<.001
Week 1	35.80 (0.590)	—	—	1.92 (.006)	—
Week 30	37.72 (0.908)	—	—	—	—

a MD: mean differences (time 2 – time 1).

b Bonferroni for pairwise comparisons.

c Repeated measures ANOVA for test of within-subjects effects.

d Not available.

## Discussion

### Principal Results

In accordance with the research hypothesis, the CHVs’ competency in providing emergency assistance and coordinating marine patient transfers after receiving this training will be statistically significantly higher than before the training (*P*<.05). The critical results indicate that there was a significant increase in the level of knowledge, skills, and competency in emergency assistance and marine patient transfer coordination from the first week after training to the 30th week.

### Strengths and Limitations

This research’s key strength is its integration of a new technology, AOC, into the training of CHVs. It also provides realistic scenarios based on community contexts gathered from community member recommendations. However, it may be limited by a lack of confidence in using the technology, which is new and exciting, among volunteers. This factor might inhibit this technology’s full potential from being realized. Therefore, CHVs’ technology competencies need to be developed regularly and continuously trained to eliminate their unconfident practice to prepare for the situations faced by people in the community. An additional constraint of this study was that participants were recruited via purposive sampling, which may limit external validity and introduce selection bias. Therefore, future studies should consider random, stratified, or cluster-based sampling for participant recruitment. Another limitation in this research needs to be addressed, specifically the gap between week 1 and week 30, during which no boosting activity was conducted, and it may affect their knowledge stability. Consequently, a further training program should consider incorporating booster activities via online sessions to monitor their knowledge and confidence in emergency illness assistance.

### Comparison With Previous Work

The findings of this study are similar to previous research, which suggested that continuous training in BLS and first-aid skills helps trainees maintain better skills and competencies in the long term [26,27]. The results of this study reflect the effectiveness of the proposed training program in CHVs’ continuous development, both in decision-making and practical skills, as well as their overall understanding of patient referral. This finding is consistent with a recent study by Pongtriang et al [15,23], who emphasized the importance of organizing continuous training programs and conducting long-term monitoring to encourage trainees to learn skills in-depth and maintain them for a longer period [28]. Indeed, competency levels can decrease significantly over time [29]. This result indicates that CHVs still require review and periodic knowledge updates [30] to be able to provide emergency assistance and transfer marine patients effectively. Another recent study highlighted that regularly restoring knowledge and skills helps slow down the rapid decline in competency levels that can occur after an initial training session [31]. Therefore, the findings of this study, where rehabilitation activities were held in week 30, effectively support long-term competency maintenance.

However, the study also revealed that the level of emergency assistance knowledge and skills among the CHVs was low overall. It is therefore important to raise awareness among nurses and other health officials of the need to study the context and background factors of people in their area to develop their potential [32], such as through the strategies tailored to this study. The recent study also suggests that considering the community context is an essential component in successful health development [33]. When analyzing the sample’s background, it also became evident that the volunteers’ education level was relatively low. Most participants had no experience assisting or responding to accidents and emergency illnesses. These factors contribute to insufficient knowledge and inadequate practices relevant to emergency assistance [34], making it crucial to maintain CHVs’ knowledge and skills. This could be improved through government support for regular training on BLS and similar emergency issues [35].

Although Thai government legislation mandates that development initiatives take place at least once per year [36], inequity and insufficient development persist due to Phaluay Island’s isolated location. This research finding aligns with a previous study, which identified several barriers to providing quality health care in rural communities [37] that require advanced care strategies, such as telehealth and satellite care, to close the health care gaps [38]. Our research incorporated digital technologies into a training program to continuously enhance the capabilities of the island’s community members. This development can be considered a new approach to promoting prehospital care through technological support systems [39]. An improved referral system can also be achieved by enhancing the abilities of community volunteers to support their community’s emergency care system. Using digital technology can aid communication, monitoring patient status [40], and facilitating the work of CHVs and family caregivers while transporting patients as well. Furthermore, the recent study supports that technology would be an advantage for community training in improving the competency of health care volunteers in the community [41]. Indonesia and the Philippines, similar to other Southeast Asian countries, encounter substantial challenges in marine health management, which necessitate the adoption of solutions, such as telemedicine systems [42]. In addition to technological interventions, the development of integrated national policies, including explicit legislation and dedicated funding for air and sea medical referrals, can address coordination barriers and reduce related costs [43]. Furthermore, strengthening community knowledge and attitudes through partnerships with local leaders may reduce delays in family decision-making and enhance understanding of health insurance systems [44].

Although previous studies have found the advantages of integrating technology to enhance people’s potential holistically, our study found that unfamiliar technologies are challenging for volunteers, resulting in excitement and anxiety about using the equipment, which affects confidence in practice. These insights were drawn from the low level of practice scores in the first trial. In addition, the issue of communicating emergency medical incidents to the emergency dispatch staff revealed that a majority of participants in the sample group were not confident and were anxious about communicating important and necessary information relevant to an incident [45]. Therefore, training with a clear format to practice both the use of necessary technologies and effective communication [46], using a step-by-step framework that can be followed and practiced independently [47], will be an important part of building confidence in developing first-aid skills and communicating more effectively in emergency confrontations [48]. When considering the knowledge and skills development program of this research, which utilized the concept of situated learning theory [22] as a framework for organizing activities to develop volunteers, the theory focuses on learning in collaborative learning situations through team participation. It is considered a highlight that is consistent with the situation and learning the context [49] of the group to understand its limitations, including support and resources for assistance at the scene, leading to problem solving and helping patients and injured people with the realistic context and situation [50], such as applying natural tools or materials in the vicinity to immobilize and move, among others. Addressing this situation is particularly important for areas with limited access and insufficient health support [51]. The findings of this development are important for the further development of training programs to enhance the knowledge and skills of volunteers in the area, which are tailored to the local context and leverage technology connectivity. This encouragement enables tracking and support for people and caregivers during the transfer, ensuring the safest possible outcome.

### Implications for Practice

Health care providers play a crucial role in establishing community training, which necessitates consideration of community context through community engagement, such as the limitations of a local health care system, the level of digital literacy of community residents, the level of social participation, and the issue of community needs of competencies development to effectively enhance the abilities of CHVs and develop an appropriate training program to maintain their knowledge and skills. In addition, due to its remote island location, similar to our study site around the world, there are several limitations to health care, support, and access to resources. With these limitations, community nurses and health providers could play an advocacy role by supporting the local people’s need for emergency care system support with community health data and presenting to higher government agencies to encourage more attention to establish a policy-driven approach to health care accessibility [52]. In addition, the result of this study could be scaled up via provincial health offices to strengthen island-based emergency medical services in southern Thailand.

### Conclusions

This study presented comparative findings on the competence, knowledge, and skills of local CHVs in providing first aid for emergency illnesses, accidents, and marine transfers of patients in a remote island area. This training program was developed based on the context, limitations, and stakeholder needs of Phaluay Island in southern Thailand. The training program utilized various methods that CHVs employ to prevent illness and attend to emergencies among the public. The findings revealed that the competence development program, which integrated digital technology to communicate with hospitals and monitor patients during rescue and transport to the destination hospital, can improve the CHVs’ knowledge and skills in terms of assessing patient condition, requesting help in an emergency, communicating effectively, BLS, and AED use. The training can also assist with coordination and practices during patient transport for effective initial care. The results further demonstrated that the application of situated learning theory, the community context, and technology integration, with appropriate components, can improve the CHVs’ competence and learning outcomes. However, to support the continuity and sustainability of competence in the long term, it is necessary to study development strategies that follow the changing context and relevant community factors. This may improve access to emergency medical services for local people and reduce the likelihood of disability and subsequent mortality.

## Abbreviations

AED: automated external defibrillator
AOC: ambulance operation center
BLS: basic life support
CHV: community health volunteer
CVI: content validity index
